# Hospital and Institutional News

**Published:** 1914-07-18

**Authors:** 


### HOSPITAL AND INSTITUTIONAL NEWS.

### PERTH ROYAL INFIRMARY.

Following upon their visit to Glasgow and their visits to three leading institutions there, the King and Queen on July 10 paid a visit to Perth and opened the new buildings of the Royal Infirmary there. The old infirmary in York Place was opened in 1838, and was altered and added to as years went on. Finally, in 1909, a decision was taken to rebuild on a new site in the Glasgow Road. The buildings and grounds extend over eleven acres, and the total cost, including that of the children's block, is estimated at £49,700. The children's block, it may be added, contains two large wards, one of which has been erected as a King Edward VII. memorial out of funds collected for that purpose; and the other by the Messrs. Whyte as a memorial of their father. There are also separate kitchen, admission, and administrative blocks, as well as a medical and a surgical block. In all the new infirmary accommodates 146 patients, and by the use of brick and rough-cast for the exterior, the cost has been kept down to about £340 per bed. The architect is Mr. James Miller, F.R.I.B.A., Glasgow. Mr. C. A. Murray, of Taymount, the chairman, has presented the equipment of the *x*-ray room.

### A MUNIFICENT COMMEMORATION OF THEIR MAJESTIES' GLASGOW VISIT.

In commemoration of the visit of the King and Queen to Glasgow, Lord and Lady Newlands are giving £25,000 to complete the endowment of the Lady Hozier Convalescent Home at Lanark, an appanage of the Western Infirmary, Glasgow. This home was built and equipped in 1891 by the late Lord Newlands (then Sir William Hozier), in memory of his wife. It was opened in 1892, and is said to have cost nearly £8,000. Three years later Sir William Hozier invested £10,000 in the names of trustees as an endowment fund towards the maintenance of the institution. In 1898 he was raised to the peerage as Lord Newlands, and was succeeded in 1906 by his son, the present peer. The latter has continued to take a deep interest in the welfare of the home, and last year made a donation of £516 to wipe out an adverse balance on the year's working. At present the endowment fund stands at £12,600; most of this is, of course, Sir William Hozier's original gift, and £2,000 more came from the estate of the late Mr. James Dick. The home accommodates forty patients. Last year there were admitted 367 males and 213 females from the Western Infirmary, who resided on an average a fortnight each. To have the financial burden of their Convalescent Home entirely lifted from their shoulders must be a great boon to the finance committee of the Western Infirmary; and the heartfelt thanks, not only of this institution, but of all Glasgow will be rendered to Lord and Lady Newlands for their truly regal benefaction.

### THE DUNDEE CHILDREN'S HOSPITAL.

An unfortunate hitch has taken place with regard to the progress of the Children's Hospital which is being promoted in Dundee. Mr. Alexander Keiller, of Morven, had offered to present a site for the institution in Perth Road, to the east of Binrock. The directors of the Infirmary, however, have now reported to the Lord Provost of the city that this site is unsuitable for the purpose, a condemnation which outside opinion does not unanimously support. The findings of the directors are, however, quite definite on the point, and it is interesting to note that a special visit was made to Glasgow to learn as much as possible from inspection of the new Children's Hospital at Yorkhill (see The Hospital, July 4, p. 379). It is considered that for the time being a hospital to accommodate 100 children under twelve will suffice. The cost (exclusive of site) of building, equipping, and furnishing a first-rate hospital is estimated at about £40,000, and annual maintenance at £6,000. The site offered by Mr. Keiller is judged by the directors and by the visiting medical staff to be barely adequate in size, and, owing to the conformation of the ground, to militate against the erection of suitable buildings. The directors advise, in preference, that a site in the near neighbourhood of the Infirmary should be obtained. Inquiries for such a site have been made, but they "are not yet in a position to report thereon." It is also suggested that a minimum capital sum of £100,000 should be raised before the scheme is proceeded with, to provide an endowment fund as well as a building fund. Towards this sum about £18,000 has already been subscribed, made up of £10,000 from Mr. F. B. Sharp, £5,000 from Mr. and Mrs. Gilroy, and £3,000 from Lady Ogilvy Dalgleish.

### THE MASSACHUSETTS GENERAL HOSPITAL.

We publish this week (pp. 439-42 and p. 444) three separate articles dealing with different aspects of one of the most progressive and up to date of American institutions, the Massachusetts General Hospital, Boston. It is hardly necessary to commend these articles to the very careful attention of hospital administrators, medical staffs, and architects; for they carry with them their own recommendation, and the indelible stamp of distinction which marks out this fine hospital is obvious in the accounts which we now publish. Institutional methods in the United States are not always the same as those to which we in this country are accustomed; nor can they ever be the same, since the environing conditions are in some respects fundamentally distinct. The financial problem, notably, is a very different thing in the two cases; and there are many other divergences. But notwithstanding this, and making all allowances where necessary, there is much in the American system, as exemplified in the best hospitals, which might be emulated, or at least adapted, in this country. The institutional spirit, in particular, is nowhere exhibited more loftily and more abundantly than in the front-rank hospitals of the United States and of Canada; and the articles to which we refer help to show how it is fostered and cultivated by wise guidance and perfection of organisation.

### THE ROYAL DEVON AND EXETER HOSPITAL.

The management of the Royal Devon and Exeter Hospital are appealing to the subscribers and the general public for £10,000 for necessary improvements. During the past year they have already provided out of capital a special department for ear, nose, and throat diseases, an accident department, and two special observation wards, but there are further urgent needs to be faced, and as soon as possible. First comes a special wing to provide proper accommodation for the nursing staff. The astounding confession is made that at present day and night nurses have "occasionally" to share the same bed, on the Box and Cox principle. How the committee can have allowed themselves to spend money on special departments while this disgraceful state of things was going on we cannot comprehend. Still, it is a step forward that they are seeking funds to remedy it. Then, next, the remodelling of the electrical department is desired. This was originally created and equipped at the expense of Mrs. E. A. Sanders, of Stone House, and the work of enlarging and extending it is being in part defrayed out of the further gift of £500 from the same lady. The purchase of radium is also put forward as one of the hospital's requirements, but we trust that not a penny will be thus expended until a proper nurses' home has been provided, and the present stigma upon the institution thereby removed.

### A MANCHESTER BABIES' HOSPITAL.

The success of the Infants' Hospital in Vincent Square has evidently been observed outside London, for it is announced that Manchester is to have a similar institution. A committee of nine Manchester women doctors have decided to open a small hospital in Clarendon Street, Chorlton-on-Medlock, for babies suffering from gastro-intestinal disorders or defects of nutrition, and a preliminary appeal for funds has been issued. It is proposed to open early in August, in order to meet the special needs of the hot summer months, and Miss Margaret Ashton has given £300, with the proviso that the expenses for opening on August 1 are secured by that date. It is expected that the first year's work will cost about £800, so it is clear that the new hospital is beginning in a very modest way. The following are the women doctors who are promoting the enterprise: Elsie Brown, M.B.; Catherine Chisholm, M.D.; Catherine L. Corbett, M.B.; Gertrude H. Geiler, M.D.; Mabel May, M.B.; Adelaide Renshaw, M.B.; Florence Robinson, M.B.; H. G. Saul, M.D.; Vera Weitzmann, M.B.

### NEW ACCIDENT HOSPITAL AT MANCHESTER.

When the removal of the Manchester Infirmary from its old site in Piccadilly was decided upon, it was also determined to build and maintain a central branch for accident cases as near the old site as possible, as mentioned in The Hospital, July 4, p. 367. The accident wards are all that is left of the old infirmary, having been kept intact until the new central branch should be ready. The latter is now practically complete, and will be opened for the reception of patients in a few weeks. The Piccadilly site will then be completely cleared by the demolition of the buildings still standing there. The new building has been erected upon the site of the historic Roby Chapel and Schools, only a stone's throw from Piccadilly itself. The cost of building and equipment is about £25,000, towards which £18,000 had already been received at the date of the last annual meeting of the infirmary. The new infirmary itself, it will be remembered, was opened entirely free from debt.

### DETAILS OF THE ACCOMMODATION.

On the ground floor are out-patient rooms on the most approved lines, and an accident-room with two observation-wards of two beds each; also the *x*-ray room and the operating theatre. Further, there are a committee-room, the house surgeon's room, offices, and an isolation room for temporary accommodation of infectious disease pending removal. On the first floor are four wards accommodating ten patients each, with necessary bath and sink rooms and lavatories, also sitting and dining rooms for the nurses, and the kitchen, pantry, and larder. The second floor is occupied by bedrooms for doctors, nurses, and servants. The floors throughout are of terrazzo, covered by linoleum, and the walls in all patients' departments, lavatories, and bathrooms are tiled. The building is warmed throughout by hot water on the "Reck" system, and the sterilising and part of the cooking will be carried out by steam.

### THE BRITISH HOSPITAL FOR MOTHERS.

The amalgamation of the British Lying-in Hospital, lately in Endell Street, with the Homes for Mothers and Babies in Wood Street, Woolwich, under the above new title, appears (as explained in The Hospital, July 4, 1914) to have elicited the remonstrance of the London County Council on the ground that the removal of the former institution to Woolwich will shift the area of hospital benefit. In the middle of the eighteenth century the failure of certain subscribers to the Middlesex Hospital to secure the allocation of subscriptions given towards the up-keep of the lying-in ward in that institution to that specific purpose, induced them to establish a special hospital by public subscription in Brownlow Street, Long Acre, that locality being chosen as midway between the Cities of London and Westminster, the wants of which it was doubtless intended to supply. This Lying-in Hospital for Married Women was opened for the reception of patients in December, 1749

(the title "British" was adopted in 1756), and exactly a century later it was removed to Endell Street. No provision was made for the delivering of patients at their own homes, but shortly after its foundation female pupils were received for training as midwives. The choice of Endell Street for the site of the second building tends to support the argument that the removal to Woolwich would not be in accordance with the intentions of the original promoters. On the other hand, the same remark would apply to the removal to a different locality of any of the earlier voluntary hospitals, while the altered methods of transit make light of comparative distance.

### INCORPORATION OF GREAT ORMOND STREET HOSPITAL.

At a Special Court of Governors on July 15, certain resolutions were submitted for confirmation which had previously been passed at a Special Court on May 20. These resolutions provide for the incorporation of the hospital under the Company Laws as a company limited by guarantee. It was the original intention of the Governors to petition the Privy Council for a Royal Charter, as so many other hospitals have successfully done during the last few years. The Senate of London University, however, raised objections, and the Privy Council hung the matter up until it was seen what steps would be taken to put into effect the recommendations of the Royal Commission on the University. As there was no immediate prospect of any such steps being taken, it seemed advisable to the committee to seek incorporation and afterwards, if thought necessary, to proceed with the petition for a Royal Charter. The advantages in legal status derived from a charter (or from incorporation) are so great that they are becoming more and more widely recognised and sought after. As a rule a Royal Charter is regarded as preferable to incorporation; for it definitely protects the charity from absorption by the State against the wishes of the managers. But both courses are equally advantageous by enabling the hospital to sue (or be sued) without any possibility of arguments as to the validity of the action taken, or of the individual as opposed to the corporate liabilities of the governors, secretary, or medical staff.

### HOSPITAL EXTENSION AT WILLESDEN.

In the last five or six years the not long ago semi-rural district of Willesden has been rapidly built over, and is now to all intents part and parcel of Greater London. It is not surprising, therefore, that the Willesden Cottage Hospital managers are finding the accommodation at their disposal inadequate to meet the calls upon it, and are preparing for the inevitable extension. At a meeting of the Council early in July, a report of the Finance and Building Enlargement Committee was unanimously adopted, recommending the erection of a hospital with fifty or sixty beds, staff accommodation, and so forth, at a cost of £12,000. Competitive designs are being sought for, and £100 is to be raised to provide prizes in connection with the competition. The scheme includes an out-patient department, a resident medical officer, and a casualty department, all of which adjuncts, very necessary and desirable in themselves, will tend to make the name of "Cottage Hospital" misleading and undignified. We advise the Council, when they make their appeal for the funds wherewith to build, to drop the word "Cottage" from the name of their institution.

### A CURIOSITY OF A HOSPITAL.

On June 21 a young man at Castleford was certified to be suffering from small-pox, and was immediately ordered to be removed to the Glass Houghton Small-pox Hospital, maintained by the Normanton and District Isolation Hospital Committee. Now this was the first time the small-pox hospital had been required since its erection, and even then it was only in use a few days, for the medical superintendent diagnosed the case as severe chicken-pox, and the patient was discharged on June 30. However, the commissioning of the hospital for those few days revealed conditions which Dr. Hartley thought it necessary to report to the Committee, and his report has aroused considerable local interest. The building is designed for half a dozen beds, so that it would not cope with an epidemic of even a most moderate dimension. In the building there is no sink whatsoever, and no drainage. The sanitary conveniences are totally inadequate, and there is no water supply. Water is carried to the hospital once a week in a water-cart. Under the tap of this contrivance a tin is placed to catch the drip, and Dr. Hartley once saw a dog refreshing his thirst therefrom. There is no light, except from candles and lamps. The heating is provided by stoves, dangerously situated on wooden floors, with hot chimneys passing through wooden walls and a wooden roof! Two fire extinguishers exist, which could only put out a fire at the risk of asphyxiating the patients. There is no telephone whereby to summon help. There are no means of disinfection, except the bath. Probably tramps are accustomed to sleep in the "hospital," for one night the door was tried by a wanderer of this sort.

### TWO BOURNEMOUTH INSTITUTIONS.

At the Royal Victoria and West Hants Hospital, Bournemouth, some recent improvements have been carried out which will be much appreciated by the nursing staff. A large house has been purchased next door to the Victoria branch of the hospital, to serve as a nurses' home for that branch. Nearly every nurse, it is stated, has a separate bedroom, and the garden and recreation rooms are expected to prove a great boon. At the Boscombe branch a new administration block is nearly finished, and will enable the same policy of greater comfort for the nursing staff to be carried out. Further, the whole scale of wages has been revised throughout the hospital, together with a more generous provision for uniforms. We congratulate the Board on this generous line of treatment, which is sure to result in increased efficiency. At another Bournemouth institution, the Royal National Sanatorium, the opposite policy seems to be in favour. Hitherto the Sanatorium has been nursed by three sisters, two staff nurses, and five probationers; but in future there are to be seven probationers and no staff nurse. Not only so, the salaries of the sisters are to be cut down, and also those of the probationers. The committee, in the memorandum (accompanying the Annual Report) in which these changes are announced, do not give any reason for them; and in view of the fact that there was a surplus on last year's working of £986 we have grave doubts as to the wisdom of their policy, let alone of its justice. The other important feature of the Report is the resolution carried at the Annual General Meeting on June 10 after a long discussion. It is to the following effect: That, in the opinion of this meeting of the Governors, it is urgently desirable that immediate steps be taken for the removal of the Sanatorium to a larger and better site in the country; that the local committee be instructed to prepare a scheme and to report thereon to the Governors as soon as possible; and that a special meeting of Governors be convened for the purpose.

### THE MANCHESTER RADIUM FUND.

Only a fortnight ago a brief comment was made in these columns (The Hospital, July 4, 1914, p. 367) on the public appeal for subscriptions to a central radium fund for the Manchester hospitals. Since then the local press has taken the matter up very warmly; the *Daily Dispatch*, *Evening Chronicle*, and associated newspapers controlled by Messrs. Hulton, have started auxiliary funds which are mounting into thousands of pounds. On July 15, the total sum at the disposal of the Manchester and District Radium Committee amounted to over £20,000; and all kinds of schemes are being promoted to increase it materially. The authorities have already set about the business of organisation, in readiness for the delivery of the first instalment of the radium ordered for October. Mr. Hartley Lupton, of the Physical Laboratory, Manchester University, has been appointed physicist in charge of the radium department at the Manchester Royal Infirmary; and Dr. Marsden, also of the Physical Laboratory at the university, is to be consulting physicist for six months, while the radium is being standardised and the laboratory equipment got into order. Both officers will take up their appointments at the middle of September.

### A QUESTION OF RESIDENCE.

A report of the Asylums Committee of the London County Council raises an important problem with regard to residence of officials in institutions. The new Maudsley Hospital is now in course of erection, but the site is not large enough to enable a house to be built for the medical superintendent, and it is suggested that the superintendent should be able to find a residence for himself in the vicinity. Actual residence on the premises will not really be necessary as there will be two resident medical assistants. The Act of Parliament, however, insists that the chief officer shall reside on the premises, and the Board of Control has pointed out that no alteration can be made without Parliamentary authority. In the circumstances the committee suggests that Parliament shall be asked to make an exception of the case of the Maudsley Hospital.

### THE NEW OFFICERS OF THE ROYAL COLLEGE OF SURGEONS.

At a meeting last week of the Council of the Royal College of Surgeons Sir Watson Cheyne, Bart., C.B., was elected President, in place of Sir Rickman Godlee. Thus Lister's trusted lieutenant succeeds in the chair of the Royal College to Lister's nephew. The new President is so well known both in medical and lay circles in London that commendation of this appointment is superfluous. Sir Watson is such an exceptionally excellent speaker and so full of the Scots pawkiness that is popular in England that the success of his term of office is already assured. Sir Frederick Eve and Sir Anthony Bowlby were elected Vice-Presidents; and a series of lecturers for the ensuing year were also appointed. The Hunterian "Professors" for the year are Professor Keith, Mr. Rupert Farrant, Mr. Sampson Handley, Mr. J. H. Evans, Mr. F. C. Pybus, and Mr. Harry Blakeway. The Arris and Gale lecturers are Mr. F. Wood Jones and Mr. David Waterston. The Erasmus Wilson lecturer is Mr. S. G. Shattock; the Arnott demonstrator is Professor Keith; and the odontological demonstrator is Mr. J. F. Colyer.

### A HEALTH CENSUS IN BRADFORD.

At a meeting, on July 8, of the Health Committee of the Bradford Corporation, a novel recommendation to the City Council was adopted. The proposal which the Council is to be asked to sanction is the undertaking by the committee of a "health census" of the city at a cost not exceeding £500. If the Council approve of the suggestion, the work will be carried out by the committee in conjunction with a statistician, Professor Arthur L. Bowley, of Reading, who will advise on the scheme, and will tabulate and report upon the information gathered. The Board of Guardians, the Charity Organisation Society, and committees of the Council whose officials are versed in the making of such inquiries are being asked to allow those officials to assist the committee in the work in their overtime.

### THE BIRMINGHAM BRICK LEAGUE.

A sale has lately been held in aid of the building fund of the children's hospital now in Broad Street, Birmingham. The new institution is to be in Ladywood Road, and the foundation-stone was laid in July last by H.R.H. Princess Louise. The sale was held in connection with the Children's Brick League, which was formed last October. As the *modus operandi* of a brick league may not be generally known, it may be well to explain that any child on whose behalf one guinea is subscribed, or who gets together that sum by his or her own exertions, acquires the privilege of laying a brick inscribed with the initials of the young bricklayer. The idea evidently caught on in Birmingham, for four hundred and eighty-six children have laid their own bricks. Thirteen were laid by children from private schools who had been chosen from among their companions as representatives; and sixty-eight were laid by children attending council schools. The building has got so far that by this time, it may be supposed, no more initialled bricks can be laid; the purpose of the sale was to keep alive the sympathy which the Brick League had engendered. Institutional organisers faced with rebuilding schemes might do well to bear in mind the success of the Birmingham Brick League.

### THE CONTROL OF NURSING HOMES.

The London County Council General Powers Bill this year includes those powers of control over nursing homes which excited some comment in nursing circles last autumn. Dr. Hamer, medical officer of health for the county, has been giving evidence in support of this part of the Bill before a committee of the House of Commons. He stated that about fifty private nursing homes exist in London, most of them kept by certificated midwives, for the purpose of receiving midwifery cases at exorbitant fees, and with the greatest secrecy. In certain cases the children born in these institutions are adopted through the agency of the proprietors, and it is impossible to trace their future. It is, of course, common gossip that such places exist, but the figures given as to the number of them are distinctly astonishing.

### THE NURSES' REGISTRATION BILL AGAIN.

It has been pointed out recently that the protest against the Nurses' Registration Bill which has been sent out by Lord Knutsford has been signed by 457 matrons of hospitals, by 50 assistant-matrons, by 2,607 nurses, by 433 probationers, by 190 medical men, and by 43 laymen. One hundred and sixty-six matrons replied to this protest in favour of the Bill, while 19 are neutral. Out of these 166, 22 are matrons of Poor-Law infirmaries, or of M.A.B. hospitals. It is being stated by the promoters of the Bill that 500 matrons have signed in favour of it. The list of such names has not been available so far, and if any weight is to attach to these signatures a list of them should be published without delay, as it is held by opponents of the Bill that many of these names must be those of ex-matrons.

### THE FEEDING OF HOSPITAL NURSES.

The *Daily Telegraph* has given publicity to a letter from a layman who asseverates that hospital nurses in certain unnamed institutions are not properly fed. The complaint, he says, is not usually that the food is insufficient, but that it is badly cooked and roughly served; and he protests indignantly against such a state of affairs. According to our judgment, there is substance in these accusations, though, happily, less than there used to be: even in the last ten years there has been a notable improvement in the food provided for hospital nurses. To some extent this may be ascribed to the adoption by so many hospitals of systematised accounts on the Uniform System, whereby the actual cost of feeding the nurses is accurately ascertainable. As far as our experience goes we should say that insufficiency of food for the nurses, in quantity or quality, is still met with in a small proportion of hospitals, and that complaints as to unattractive service would be justifiable in a considerably larger percentage. With the *Daily Telegraph's* correspondent we agree that such a state of things is "utterly wrong," and that it should be reformed.

### NORWICH FOOTBALLERS AND THEIR HOSPITAL.

For eleven years past the Norfolk and Norwich Hospital has been supported by the footballers of the city through an organisation known as the Norfolk and Norwich Hospital Cup Committee, which has contributed to the hospital since its formation no less than £1,640. This year the committee has been able to make a grant of £140, and a deputation, headed by Mr. Leathes Prior, waited on the board of management to make the presentation. Mr. Prior said the movement was initiated by a working-man (Mr. Smith), and had been from first to last a working-man's movement. It was carried out by a committee of working-men, and the working-men of Norwich supported the fund by their entrance money to see the football. He also acknowledged the help of the directors of the Norwich City Football Club. Mr. F. W. Scott, one of the members of the committee, was nominated a life governor of the hospital in accordance with the rule applying to all donations of £100 or more.

### A NOVEL METHOD OF RAISING FUNDS.

It is difficult to say how far one is justified in taking the law into one's own hands, but the following story, which was recently told to a correspondent by a lady who vouches for its truth, should be of interest to institutional workers:—An individual of comfortable means, who was in the habit of frequenting a certain tea-shop, was suspected of leaving on occasions without having discharged the bill. A watch was kept, and when suspicion was made certainty the individual thus detected was requested to go to the manager's room, and was then given the alternative of defending an action in court or drawing a cheque in favour of a local infirmary. An institution in the near vicinity of the tea-rooms was shortly afterwards surprised at receiving a handsome anonymous donation.

### THE UNALLOTTED PANEL FUND IN LONDON.

The prolonged controversy over the distribution of the huge fund which has been accumulated by the London Insurance Committee owing to the failure of many thousands of the insured to choose a panel doctor has taken a new turn. A body has sprung into existence called the Panel Practitioners' Political Union, and the first aim of this alliterative society is to secure the distribution of the surplus fund among the panellers. A meeting of the new union was held on July 2 at Caxton Hall, at which the matter was debated in a very combative vein, and it was decided to follow the advice of Dr. Addison, M.P., and to send a deputation to the Chancellor of the Exchequer on the subject. One of the speakers confessed himself an ardent Tariff Reformer, another was equally attached to Free Trade; but both sank these political differences in their desire to promote the interests of the panellers' Union. Over the point now at issue there is, as we have formerly urged, something to be said on both sides. It is true, certainly, that many of those who have caused the trouble by neglecting to go on a panel list are those who have hitherto been healthy: their premiums should therefore go to reward the doctors as a makeweight against the attendance on those who are less healthy. But, on the other hand, many of the insured have not chosen a doctor because they prefer to be attended by non-panel practitioners and to pay their own charges. To the medical benefit of these the panel men have no legitimate moral claim, though they have, it is true, one based upon a promise made by the Chancellor at a time when the future of his Act seemed to be in jeopardy. The difficulty is that no one can possibly determine in what proportion these two classes of the insured exist; and this means that it is impossible to know what proportion of the surplus fund is rightly the property of the panellers. The further difficulty of adjudging how the fund should be divided is one which seems equally incapable of fair and righteous solution.

### PROBLEMS ARISING OUT OF SCHOOL INSPECTION.

A recent case in the Gloucester County Court well illustrates certain points which have been brought out in our series of articles on the school doctor's difficulties. A parent sued Dr. J. Middleton Martin, medical officer of health for Gloucestershire and school medical officer to the County Education Committee, for £6 15s. damages on the ground that without the parents' consent he had cut off a portion of the child's hair. From the evidence it appeared that the parents of this child had been warned that their daughter's hair was verminous, and that action would be taken were its condition not improved. In consequence of a hairdresser's report no steps were taken, whereupon a further warning was sent, and a prosecution was threatened were the school nurse, whose report led to this warning, not satisfied of its improvement. The parent then took the child to a medical man, who certified that, while scurfy, the child's head was not verminous. On this the parent told Dr. Martin he would not allow him again to examine the child, and that he should not send her back to school but would run the risk of proceedings. On her non-appearance at school Dr. Martin called, to satisfy himself whether the other doctor's report or that of the school nurse was correct, and cut off a portion of hair for examination. Dr. Martin stated that he asked permission before doing so. The plaintiff was awarded a farthing damages. Now this is typical of the kind of difficulties which arise in school work, and which only the greatest tact on the part of school medical officers and nurses can avoid. The verdict and the damages hardly indicate whether or not the jury thought that the school doctor had exceeded his prerogative.

### LONG-SERVICE MEDALS FOR NURSES.

Since medals of any kind are by convention, in this country, worn only by uniformed persons, there is nothing in the idea of medals for nurses which clashes with custom or tradition. Some nursing co-operations do, indeed, provide their members with badges, which are to all intents and purposes medals; but institutions have been, with exceptions, shy of adopting the custom. It is now announced that the Board of Management of the Royal Hospital for Incurables, Putney Heath, have decided to present bronze and silver long-service medals to the members of the nursing staff. It is not stated what periods of continuous service will be necessary to qualify for these medals, but they may be presumed to be fairly lengthy ones. Provided every care is taken not to create any colourable imitation of the military medals which a small percentage of the nursing profession wears, we see no reason for disapproval of this system, and some excellent reasons for praise of it. If other institutions should follow the example thus set, it would be well that some uniformity should be arrived at in the matter of the length of service necessary to qualify for long-service medals of this sort.

### THIS WEEK'S DRUG MARKET.

A fairly quiet tone prevails in the drug market, but considering the season there is not much to complain about. Opium is dearer, partly in consequence of unfavourable weather in the producing districts at the time of the ingathering, and partly as the result of an improved demand for the drug; morphine and codeine are also dearer. The general view is that lower prices are not to be expected at present. The upward movement in the price of buchu leaves is maintained. Essence of lemon is lower in price. Castor oil is slightly cheaper. Citric acid, tartaric acid, and cream of tartar continue in good demand, the tendency of prices being upward. Quotations for salts of mercury are unchanged, but the market for quicksilver is still unsettled. Cloves are dearer. Cocaine is unchanged in price, but it is thought that the low values cannot prevail indefinitely; it is said, in fact, that the low prices now ruling for coca leaves have induced planters to cultivate more profitable produce, and if this be correct the effect will be felt on the market sooner or later. The price of camphor is maintained, and a small advance in price would not come entirely as a surprise. Cape aloes and jalap have a somewhat lower tendency in prices. The position of quinine is unchanged.

